# Discrepancy in p16 expression in patients with HPV-associated head and neck squamous cell carcinoma in Thailand: clinical characteristics and survival outcomes

**DOI:** 10.1186/s12885-021-08213-9

**Published:** 2021-05-06

**Authors:** Lalida Arsa, Teerada Siripoon, Narumol Trachu, Sasithorn Foyhirun, Duangjai Pangpunyakulchai, Suda Sanpapant, Natini Jinawath, Poompis Pattaranutaporn, Artit Jinawath, Nuttapong Ngamphaiboon

**Affiliations:** 1 Molecular Histopathology Laboratory, Pathology, Faculty of Medicine Ramathibodi Hospital, Mahidol University, Bangkok, Thailand; 2 Division of Medical Oncology, Department of Medicine, Faculty of Medicine Ramathibodi Hospital, Mahidol University, Bangkok, Thailand; 3 Research center, Faculty of Medicine Ramathibodi Hospital, Mahidol University, Bangkok, Thailand; 4 Immunohistopathology and Special Laboratory, Department of Pathology, Faculty of Medicine Ramathibodi Hospital, Mahidol University, Bangkok, Thailand; 5 Ramathibodi Comprehensive Cancer Center, Faculty of Medicine Ramathibodi Hospital, Mahidol University, Bangkok, Thailand; 6 Integrative Computational BioScience Center (ICBS), Mahidol University, Nakhon Pathom, Thailand; 7 Program in Translational Medicine, Faculty of Medicine Ramathibodi Hospital, Mahidol University, Bangkok, Thailand; 8 Division of Radiation Oncology, Department of Radiology, Faculty of Medicine Ramathibodi Hospital, Mahidol University, Bangkok, Thailand

**Keywords:** Head and neck squamous cell carcinoma, p16, HPV, Oropharyngeal squamous cell carcinoma, OPSCC

## Abstract

**Background:**

Lower prevalence HPV infection has been previously reported in Thai population when compared with Western countries. p16 expression indicates HPV-associated oropharyngeal squamous cell carcinoma (OPSCC), but not non-OPSCC. We therefore evaluated the characteristic and association of p16 and HPV in Thai patients with HNSCC.

**Methods:**

We used immunohistochemistry and qPCR, respectively, to detect p16 and HPV DNA in archrival formalin-fixed paraffin-embedded HNSCC tissues. Patient characteristics and survival were analyzed.

**Results:**

p16 expression was detected in tumors of 72 of 662 (10.9%) patients with HNSCC and was significantly associated with higher-grade histology, advanced nodal stage, and oropharynx. p16 was expressed in 28 and 6.5% of patients with OPSCC or non-OPSCC, respectively, and HPV DNA was detected in 15.6 and 1% of patients, respectively. Using p16 as a surrogate for HPV status, sensitivities were 80 and 25% in OPSCC and non-OPSCC, respectively. Positive and negative predictive rates of OPSCC were 38 and 95%. Discordance rates between HPV and p16 were 23 and 7% in OPSCC and non-OPSCC, respectively. Overall survival (OS) were significantly longer in both p16-positive OPSCC (*p* = 0.049), and non-OPSCC (*p* = 0.003).

**Conclusions:**

Low prevalence of p16 and HPV associated OPSCC and non-OPSCC were confirmed in Thai patients. High discordance and low positive predictive rates of p16 were observed in HPV-associated OPSCC. p16 was a significant prognostic factor for OS for patients with OPSCC or non-OPSCC. Therefore, HPV testing should be performed to assess the association of HPV with HNSCC regardless of p16 expression.

**Supplementary Information:**

The online version contains supplementary material available at 10.1186/s12885-021-08213-9.

## Background

Human papilloma virus (HPV), which plays a major role in the pathogenesis and progression of head and neck squamous cell carcinoma (HNSCC), is more commonly associated with oropharyngeal squamous cell carcinoma (OPSCC) [1]. HPV infection induces many alterations in the CDK4-Cyclin D-Rb and apoptotic pathways such as upregulation of the expression of the cyclin dependent kinase inhibitor 2A (p16) as well as loss of retinoblastoma (Rb) and tumor suppressor protein p53 functions [2–4]. The HPV E6 protein forms a complex with the E3 ubiquitin ligase E6-associated protein (E6AP), and ubiquitinates the p53 tumor suppressor protein. Ubiquitination causes rapid degradation of p53, which deregulates the G1/S and G2/M cell cycle checkpoints upon DNA damage as well as other cellular pathways that respond to stress, ultimately leading to genomic instability [5]. The HPV E7 protein binds to the cullin-2 ubiquitin ligase complex and ubiquitinates phosphorylated Rb (pRb), resulting in deregulation of the G1/S phase of the cell cycle. In the absence of pRb function, the E2F family of transcription factors is released and S-phase genes are transcribed, leading to cell proliferation and increased expression of p16 [5].

Expression of p16 significantly correlates with the HPV status of OPSCC and serves as an independent prognostic factor for survival of patients with OPSCC treated with concurrent chemoradiation (CRT) [6]. In contrast, HPV-associated tumors are less frequent outside the oropharynx and are not associated with p16 expression [7–10]. Thus, the 2018 College of American Pathologists Guidelines recommend that p16 expression is a reliable surrogate marker to diagnose HPV-associated OPSCC when there is strong and diffuse nuclear and cytoplasmic p16 expression in ≥70% of tumor cells [11, 12]. Moreover, p16/HPV status is incorporated into the most recent TNM staging system of the 8th American Joint Committee on Cancer (AJCC) for OPSCC, which provides more accurate and rational prediction of survival of newly diagnosed patients [13]. In contrast, analyzing p16 expression in non-OPSCC is not routinely recommended, because p16 expression does not well correlate with the HPV status of tumors [12]. Moreover, there is no proven prognostic or therapeutic difference associated with the presence or absence of p16 [12]. Prognosis and the association between p16-expression and HPV in non-OPSCC remains controversial because of conflicting data [10, 14–17].

The frequencies of detection of HPV and p16 status of HNSCC vary because of differences among detection methods and cut-off values, different ethnicities, and the primary site of HNSCC [6, 12, 15, 18–23]. The incidence of OPSCC has significantly increased, predominantly in high-income countries, while that of oral cavity squamous cell carcinoma (OCSCC) has decreased [24]. In contrast, the incidences of OPSCC and OCSCC have remained constant in low-income countries. In 2011, head and neck cancers in Thailand were the third and fifth most common cancers in males and females, respectively [25]. OCSCC is the most common primary site and accounts for 30.7% of HNSCC. However, the etiological shift to OPSCC observed in the United States may be occurring in Thailand, although data for p16 expression and HPV status of OPSCC and non-OPSCC in Thailand are limited [26]. Though a low incidence of p16-positive HNSCCs in Thailand was recently reported, the correlation between p16 expression and HPV-associated HNSCC is unknown [9, 10, 23, 27]. Therefore, we used archived formalin-fixed, paraffin-embedded (FFPE) tumor tissue to evaluate the p16 and HPV status of patients with HNSCC with the aim to better understand their influences on patients’ outcomes.

## Methods

### Study design

Patients with histology confirmed HNSCC of oral cavity, oropharynx, larynx, hypopharynx, paranasal sinus, and known primary who treated at the Ramathibodi Cancer Center between January 2007 and December 2018 were identified through the Ramathibodi Cancer Registry. We analyzed available archival FFPE tumors using immunohistochemistry (IHC) to detect p16 expression, and polymerase chain reaction (PCR) for HPV DNA detection. Patients with nasopharyngeal carcinoma, cutaneous squamous cell carcinoma, squamous cell carcinoma of external ear canal, and insufficient/unavailable FFPE samples for p16 evaluation were excluded. Eligible patients’ medical records were reviewed for demographic information such as age, sex, Eastern Cooperative Oncology Group (ECOG) performance status, American Joint Committee on Cancer TNM staging (AJCC 7th ed.), smoking status, primary site of tumor, histological grade, and survival outcomes. Overall survival (OS) was defined as the time from date of tissue diagnosis to date of death from any cause or to the last follow-up. The status (living or dead) of each patient was cross-checked with the National Security Death Index of Thailand. The Ramathibodi Ethics Committee approved the study.

### Analyses of p16 expression and HPV-DNA

We used IHC (CINtec p16 Histology [Ventana, Tucson, AZ]) to analyze FFPE samples of patients with HNSCC for p16 expression. The sections were probed with a mouse monoclonal antibody against p16^INK4a^ (clone E6H4) using a Ventana Benchmark Ultra instrument. According to the most recent College of American Pathologists Guidelines, IHC detection of p16 is scored positive or negative if nuclear and cytoplasmic staining of > 70% or < 70% of cancer cells, respectively, is observed [11, 12]. Oropharyngeal and non-oropharyngeal carcinoma were evaluated using the same cut-off criteria.

Tumor tissues were microdissected from selected tissue blocks after matching with histology slides. DNA was extracted using a QIAamp FFPE tissue extraction kit (Qiagen, Hilden, Germany) following the manufacturer’s protocol and then stored at − 20 °C. DNA samples (250 ng) were measured using a Nanodrop (Thermo Fisher Scientific, Waltham, MA) and subjected to HPV genotyping using an AmoyDx High-risk Human Papillomavirus (HPV) Detection Kit (Amoy Diagnostics, Xiamen, China) following the manufacturer’s protocol. The kit has been designed to detect of the conserved L1 region in the HPV DNA of the high-risk HPV types 16, 18, 26, 31, 33, 35, 39, 45, 51, 52, 53, 56, 58, 59, 66, 68, 70, 73, and 82. The positive and negative controls for the PCR reaction were used according to the manufacturer instruction. The PCR results were reported as HPV types 16/18, other high-risk HPV types, or negative for detection. The limit of detection of the kit ranges from 50 to 1000 copies of HPV DNA per reaction, depending on different types of HPV. An internal control of the assay was provided for assessment of sample quality and the presence of PCR inhibitors (Supplement 1). All qPCR assays were performed using a BIO-RAD CFX96 Touch PCR detection system (Bio-Rad Laboratories, Hercules, CA).

### Statistical analysis

The study aimed to characterize p16 expression and HPV DNA status of archrival HNSCC tumors. We performed a correlation of p16 expression as a surrogate marker of HPV status in all OPSCC patients, using positive HPV DNA in FFPE by the PCR technique as a gold standard for HPV-associated OPSCC. Sensitivity, specificity, false positive, false negative, positive predictive rate, and negative predictive rate of p16 expression as a surrogate marker of HPV-associated tumors were determined.

Categorical data are expressed as numerals and percentages. Differences in proportions were analyzed and compared using the chi-square or Fisher exact test, as appropriate. Continuous variables were summarized using descriptive statistics and compared using the Student *t* test. Survival analyses were performed using the Kaplan–Meier method, and the survival curves were compared using the log-rank test. A significant difference is indicated by *p* < 0.05. Statistical analyses were performed using the Statistical Package for the Social Sciences (SPSS) version 20.0.

## Results

### Patient characteristics and p16 expression

**Fig. 1:**
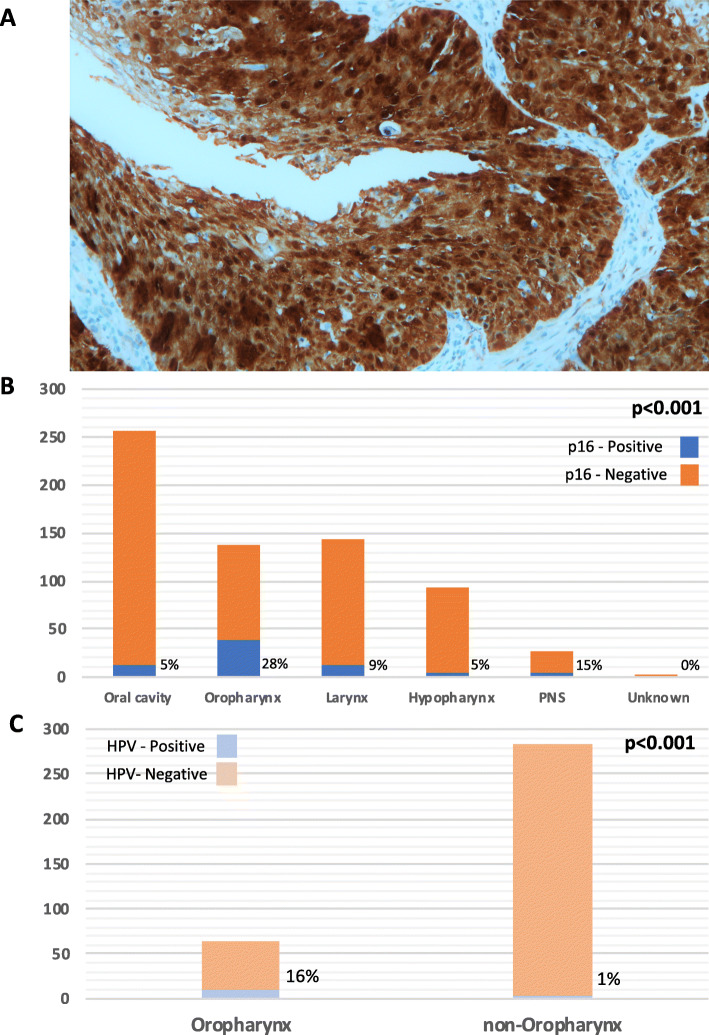
p16 immunohistochemistry in the FFPE section of tumor from tonsil shows diffuse strong nuclear and cytoplasmic positivity for p16 in more than 70% of tumor cells. The tumor is also positive for HPV DNA PCR (methylene blue counterstaining, original magnification × 100) (**a**). Frequencies of detection of p16 and HPV according to primary tumor site (**b** and **c**)

We detected p16 expression in 72 of 662 (10.9%) tissues of patients with HNSCC. An example of p16 positive case was shown in the Fig. 1a. Most patients were males and smokers presented with stage IVa/b disease (Table 1). The baseline characteristics of p16-positive and p16-negative patients were comparable, except for the site of primary tumor, histological grade, and lymph node (LN) stage at diagnosis (Table 1). p16 expression was significantly associated with higher-grade histology (*p* < 0.001) and advanced LN stage (*p* = 0.049). The oropharynx was the primary site of tumors that were significantly associated with p16 expression (*p* < 0.001). Patients with p16-positive tumors underwent definitive CRT more than p16-negative patients (55% vs 31%), whereas surgery alone was more preferred in p16-negative patients (54% vs 32%; *p* < 0.001). The percentage of p16-positive tumors according to each primary tumor site is shown in Fig. 1b. The frequencies of detection of p16-positive HNSCC tumors were the oropharynx, 28% and non-oropharynx, 6.5% (paranasal sinus, 15%; larynx, 9%; oral cavity; 5%; and hypopharynx. 5%). Among patients with non-OPSCC HNSCC, there was no significant difference in baseline characteristics between p16-positive and p16-negative patients (Supplement 2).

**Table 1:** Patient characteristics

	p16-positive ***n*** = 72 (%)	p16-negative ***n*** = 590 (%)	***p***-value
Median Age (years, range)	58 (38–84)	62 (20–95)	0.252
Age ≥ 65	24 (33)	242 (41)	
ECOG			
0–1	67 (93)	532 (90)	0.528
≥ 2	5 (7)	58 (10)	
Sex			
Male	53 (74)	441 (75)	0.886
Female	19 (26)	149 (25)	
Smoking			
Never	28 (40)	193 (35)	0.427
Ever	42 (60)	361 (65)	
Mean pack-year (+/−SD)	23.9 (14.3)	26.3 (18.2)	0.418
Missing	2	36	
Site of primary tumor			
Oral cavity	12 (17)	245 (41)	< 0.001
Oropharynx	38 (53)	99 (17)	
Larynx	13 (18)	131 (22)	
Hypopharynx	5 (7)	89 (15)	
Paranasal sinus	4 (6)	23 (4)	
Unknown primary	0	3 (1)	
Site of primary tumor			
Oropharynx	38 (53)	99 (17)	< 0.001
Non-oropharynx	34 (47)	491 (83)	
Histology grade			
Well differentiated	7 (12)	194 (38)	< 0.001
Moderately differentiated	34 (57)	226 (44)	
Poorly-differentiated	13 (22)	37 (7)	
Undifferentiated	6 (10)	59 (11)	
Non-specific type	12	74	
Stage at Diagnosis (AJCC 7th)			
I	6 (8)	85 (15)	0.425
II	8 (11)	52 (9)	
III	11 (15)	91 (15)	
IVa/b	45 (63)	325 (55)	
IVc	2 (3)	35 (6)	
T-stage			
1–2	36 (50)	224 (38)	0.057
3–4	36 (50)	360 (62)	
Tx	0	6	
N-Stage			
0	21 (29)	261 (44)	0.049
1	14 (19)	96 (16)	
2	33 (46)	188 (32)	
3	4 (6)	44 (8)	
Nx	0	1	
Definitive treatment for locally advanced disease			
Surgery alone	23 (32)	306 (54)	< 0.001
Surgery with adjuvant CRT	9 (14)	83 (15)	
Definitive CRT	39 (55)	179 (31)	

### HPV-DNA status and association with p16 expression

Adequate tumor DNA were obtained from 348 of 662 patients. HPV DNA was detected in 14 of 348 patients (4.0%) (10 OPSCC and 4 non-OPSCC patients). Ten of 64 patients (15.6%) with OPSCC were positive for HPV DNA (8 and 2 cases for HPV types 16/18 and other high-risk HPV types, respectively). On the other hand, 4 of 284 (1%) patients with non-OPSCC were positive for HPV DNA (2 and 2 cases for HPV types 16/18 and other high-risk HPV types, respectively) (Fig. 1c). HPV status was significantly associated with p16 expression in all patients with overall HNSCC (*p* < 0.001) and OPSCC (*p* = 0.001) patients, but not patients with non-OPSCC (*p* = 0.243) (Table 2). When p16-expression was evaluated as a surrogate marker of HPV status, using HPV DNA as a gold standard, the sensitivities of IHC for detecting p16 were 80 and 25% in patients with OPSCC and non-OPSCC, respectively (Table 2). The false-negative rate of p16-IHC for patients with non-OPSCC and OPSCC were 75 and 20%, respectively. However, high-negative predictive rates of p16 indicating HPV status were 95 and 99% for patients with OPSCC and non-OPSCC, respectively. Discordance rates of HPV/p16 status were 23 and 7% for patients with OPSCC and non-OPSCC, respectively. The numbers of patients with OPSCC with p16-positive/HPV-negative and p16-negative/HPV-positive tumors were 13 (20%) and 2 (3%), respectively. Among patients with non-OPSCC, 18 were p16-positive/HPV-negative.

**Table 2:** Correlation between p16 expression and HPV status of all patients with head and neck cancers and those with oropharyngeal cancer

All patients ***N*** = 348				OPSCC patients ***N*** = 64				Non-OPSCC patients ***N*** = 284			
	HPV+ ***n*** = 14 (%)	HPV- ***n*** = 334 (%)	***p***-value		HPV+ ***n*** = 10 (%)	HPV- ***n*** = 54 (%)	***p***-value		HPV+ ***n*** = 4 (%)	HPV- ***n*** = 280 (%)	***p***-value
p16+ *n* = 40 (%)	9 (64)	31 (9)	< 0.001	p16+ *n* = 21 (%)	8 (80)	13 (24)	0.001	p16+ *n* = (%)	1 (25)	18 (6)	0.243
p16- *n* = 308 (%)	5 (36)	303 (91)		p16- *n* = 43 (%)	2 (20)	41 (76)		p16- *n* = (%)	3 (75)	262 (94)	
**p16 testing when using HPV DNA as a gold standard**											
Sensitivity			64%	Sensitivity			80%	Sensitivity			25%
Specificity			91%	Specificity			76%	Specificity			94%
False positive			9%	False positive			24%	False positive			6%
False negative			36%	False negative			20%	False negative			75%
Positive predictive rate			22%	Positive predictive rate			38%	Positive predictive rate			5%
Negative predictive rate			98%	Negative predictive rate			95%	Negative predictive rate			99%
Discordant rate			10%	Discordant rate			23%	Discordant rate			7%

### Survival outcomes

**Fig. 2:**
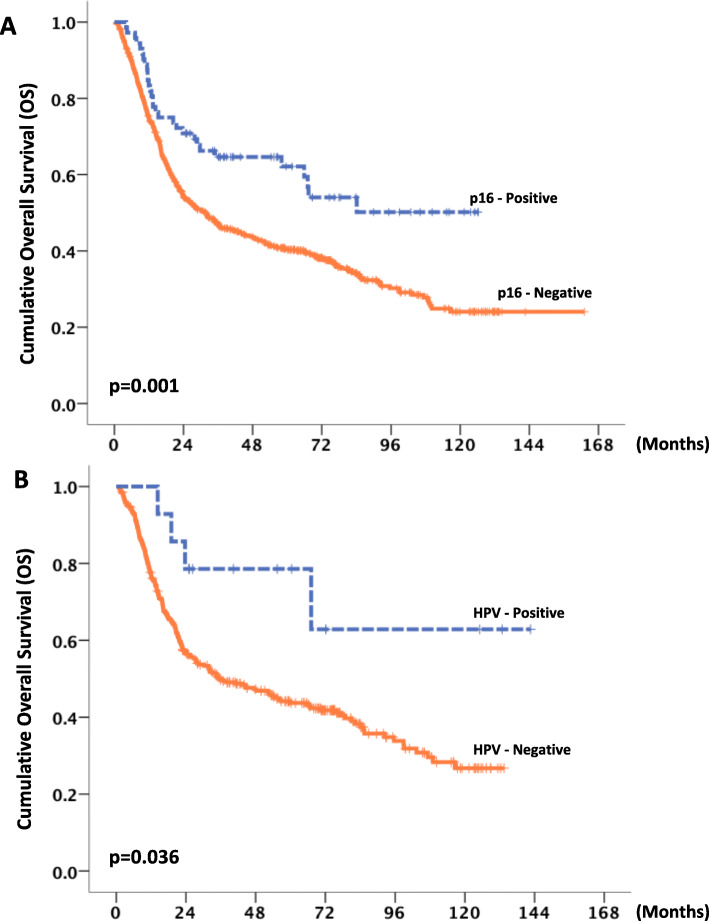
Overall survival (OS) according to p16 expression (**a**) and HPV status (**b**)

**Fig. 3:**
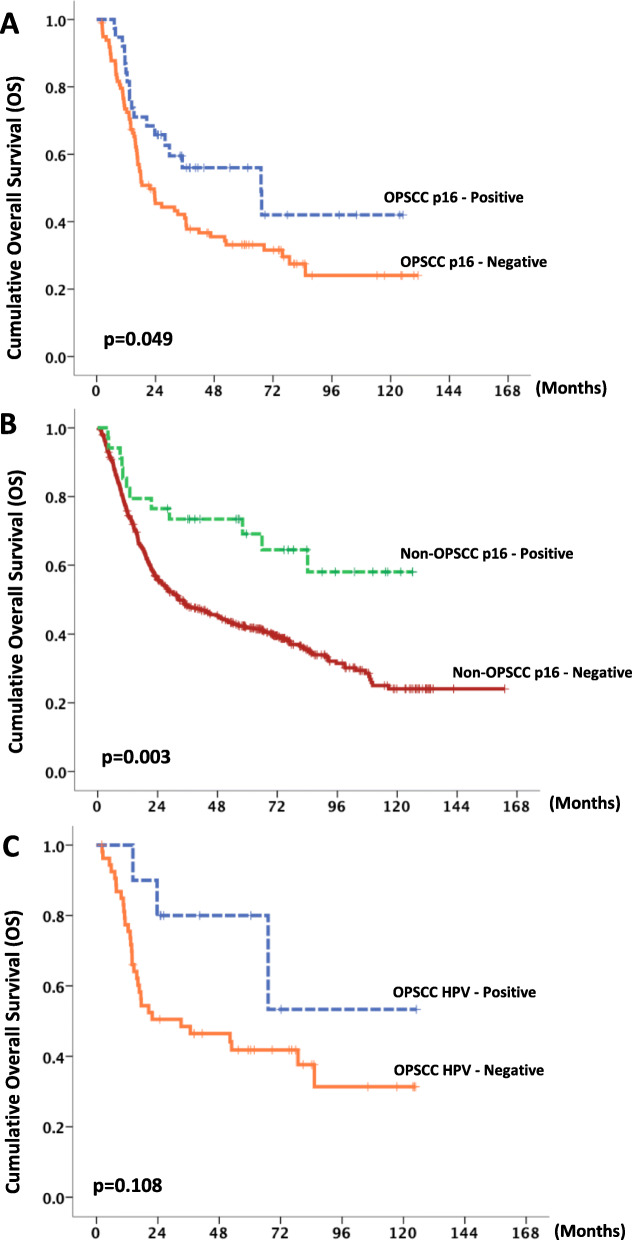
OS of patients with OPSCC and non-OPSCC according to p16 expression and HPV status

The median follow-up was 28.1 months. Patients with p16-positive were associated with significantly longer median OS compared with those with p16-negative tumors (not reached [NR] vs 31.6 months, *p* = 0.001) (Fig. 2a). Similarly, patients with HPV-associated HNSCC experienced significantly longer median OS (NR vs 35.8, *p* = 0.036) (Fig. 2b). The median OS of p16-positive patients with OPSCC was significantly longer (67.1 months vs 21.8 months, *p* = 0.049) (Fig. 3a), although there was no significant difference between HPV-positive patients (NR vs 33.1 months, *p* = 0.108) (Fig. 3c). p16-positive patients with non-OPSCC experienced significantly longer median OS compared with p16-negative patients (NR vs 32.7 months, *p* = 0.003) (Fig. 3b).

**Fig. 4:**
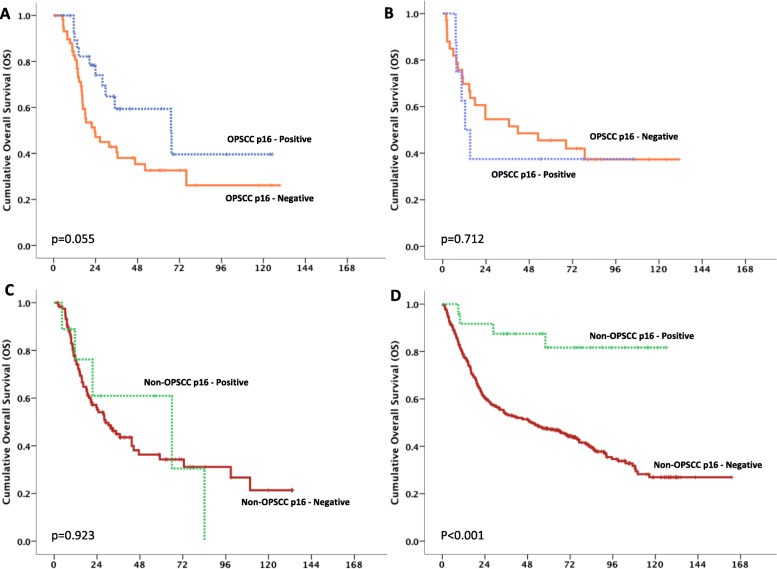
OS of HNSCC patients according to definitive treatment; patients with OPSCC treated with definitive CRT (**a**), surgery with or without post-operative CRT (**b**), patients with non-OPSCC treated with definitive CRT (**c**), surgery with or without post-operative CRT (**d**)

Survival outcomes with treatment specific modalities of locally advanced HNSCC patients are demonstrated in Fig. 4. In patients with OPSCC treated with definitive CRT, p16-positive patients had numerically longer median OS when compared with p16-negative patients (67.1 vs 23.5 months; *p* = 0.055) (Fig. 4a). There were no statistically different in OS and p16 status among OPSCC patients treated with definitive surgery with or without post-operative CRT (*p* = 0.712) (Fig. 4b), and non-OPSCC patients treated with definitive CRT (*p* = 0.923) (Fig. 4c). In non-OPSCC patients who underwent definitive surgery with or without post-operative CRT, patients with p16-positive had significant longer median OS when compared with p16-negative patients (not reach vs 50.5 months; *p* < 0.001) (Fig. 4d).

## Discussion

Low prevalence of p16 expression in HPV-associated HNSCC among Thai patients with OPSCC and non-OPSCC were observed in our study (Fig. 1a and b). Our results are consistent with those of previous studies showing that 12% Thai patients with HNSCC were p16-positive [10, 23]. Another study conducted in Thailand reported that among 504 patients with HNSCC, there was a low prevalence of p16-positive (28%) and HPV-positive (14.5%) OPSCC as well as in non-OPSCC (excluding hypopharyngeal cancer) (4% p16-positive and 3% HPV-positive) [9]. The low prevalence of HPV-associated OPSCC in Thailand was comparable with the result of a study from Taiwan [27]. On the other hand, the prevalence of HPV-associated OPSCC in other Asian countries such as Japan and China, which reported the prevalence of HPV-associated OPSCC of 32–38%, were higher than those of Thailand and Taiwan [20–22, 27, 28]. However, the overall prevalence of HPV-associated OPSCC (63.7%) and non-OPSCC (9.4%) in patients from Western countries was significantly higher compared with those in Asian countries [6, 14]. The differences in p16 positive/HPV-associated OPSCC between races may also be partly explained by the differences in oral sexual behavior and, to a lesser extent, immunogenetics [29, 30]. Differences in sample selection and methods of HPV detection among studies may partly contribute to discrepant results [18]. For example, variations in the prevalence of p16 expression in OPSCC may be explained by interobserver variation. Furthermore, some studies employed different definitions and cut-off values for p16-positivity, which are inconsistent with the recent guidelines of the College of American Pathologists for interpreting the significance of p16 expression levels [12].

A study of 388 patients with OPSCC, conducted in the Netherlands, used p16 IHC and PCR for HPV DNA, which are similar methods for HPV detection in our study [19]. This study demonstrated high consistency, with a positive predictive rate of 88% of p16 expression and HPV DNA in patients with OPSCC [19]. In contrast, our study (Table 2) observed much lower positive predictive (38%) and higher discordant (23%) rates. The discrepancy in the testing efficacy of our present study might be explained by the lower prevalence of p16-positive/HPV-associated OPSCC in the Thai population. It is possible that employing p16 status as a surrogate marker for HPV-associated OPSCC might be more effective in high prevalence p16-positive/HPV-associated OPSCC population such as in Western countries. Moreover, previous studies reported 5–20% false positive rate of p16 IHC positivity in HPV-negative OPSCC patients, in which the false positive rate tended to be higher in low prevalence HPV-associated OPSCC areas [6, 31]. Other potential molecular mechanisms might also be contributed to this discrepancy. Overexpression of p16 in the absence of HPV infection, which is not uncommon in the oropharynx, may occur through other molecular mechanisms, such as inactivation of Rb by mutations or deletion, amplification of p16, and mutations of histone H3 lysine 36 methyltransferase genes [31, 32]. On the hand, HNSCC patients with HPV DNA positive/p16-negative might carry mutations or deletions of p16 gene, which prohibits p16 protein from being overexpressed [31]. However, the present and published studies showed that p16 was an ineffective surrogate marker for HPV in non-OPSCC, although a high negative predictive rate of p16 was consistently observed [12, 14, 17].

Though the current standard methods for detection of HPV infection in OPSCC are based on detection of E6/E7 mRNA detection [33], OPSCC tumor samples were mainly obtained as FFPE tissue in which RNA might not be reliably preserved [34–36]. Therefore, in our study, we selected HPV DNA detection method by qPCR to confirm the presence of HPV infection in HNSCC. Although HPV DNA detection, either by PCR or ISH, has an advantage for feasible applications on FFPE specimens, and wide availability on many automated platforms, this technique does not distinguish between transcriptionally active and unrelated or transient HPV infection. Whether these limitations of HPV DNA detection might contribute to the discrepancy of p16 expression and HPV status in our study, further detection of HPV E6/E7 mRNA, using ISH in p16-positive HNSCC samples might be able to confirm these results.

p16 expression is a significant and consistent prognostic factor for OS of patients with OPSCC [6, 9, 17, 19]. However, numerous studies reported discrepancies between HPV and p16 status in OPSCC. Nauta et al. [19] demonstrated that patients with p16-positive/HPV-negative OPSCC had distinct features and shorter OS compared with patients with p16-positive/HPV-positive OPSCC. Similarly, a meta-analysis of HPV and p16 status of all HNSCC subtypes found that the 5-year OS of patients with p16-positive/HPV-negative HNSCC was shorter than that of those with p16-positive/HPV-positive [17], while patients with p16-negative/HPV-positive or p16-negative/HPV-negative HNSCC experienced the shortest OS [17]. We were unable to conduct an equivalent evaluation here because of the handful numbers of patients with p16-positive/ HPV-positive HNSCC and the relatively small number of subjects in the OPSCC subgroup. Recruitment of more Thai patients with OPSCC with longer follow-up times is in progress.

The guidelines of the American Society of Clinical Oncology (ASCO) and the College of American Pathologists do not recommend routine testing of patients with non-OPSCC because of conflicting data on prognosis as well as no significant differences in the outcomes of therapy [11, 12]. However, we found that p16 expression was a significant prognostic factor for OS of patients with non-OPSCC, which was consistent with the findings of the RTOG 0129, 0234, and 0522 studies and a meta-analysis of HPV and p16 in HNSCC [14, 17]. There was no difference in baseline clinicopathologic factors between patients with or without p16 expression in our non-OPSCC cases (Supplement 1), suggesting that p16 expression might be an independent prognostic factors for OS in this group of patients.

Though p16/HPV status is known as a prognostic factor for survival of patients with OPSCC, predictive value of p16/HPV for therapeutic guidance remains unclear [6, 9, 17, 19]. Since p16-positive/HPV-positive OPSCC has better survival outcomes as it is more sensitive to treatment with chemotherapy and radiotherapy, the attempt to de-intensify treatment of OPSCC to reduce toxicity and morbidity has been made [37]. Recently, two large phase III randomized studies of patients with HPV-associated OPSCC (De-ESCALaTE HPV and NRG Oncology RTOG 1016) attempted to de-intensity chemotherapy using cetuximab compared with standard cisplatin for CRT [38, 39]. However, both studies failed to demonstrate non-inferiority of cetuximab to cisplatin. In contrast, patients with HPV-associated OPSCC treated with cetuximab radiotherapy had significantly worse survival outcomes [38, 39]. Thus, concurrent cisplatin with CRT remains the standard of care for patients with OPSCC, regardless of HPV/p16 status.

## Conclusion

Our study observed that Thai patients with OPSCC and non-OPSCC infrequently expressed p16 and had low prevalence of HPV infection. Furthermore, p16 expression was a significant prognostic factor for OS of patients with both OPSCC or non-OPSCC. p16 expression was acceptable as a surrogate marker only for HPV associated OPSCC.

However, higher discordant and lower positive predictive rates of p16 expression were observed in patients with HPV-associated OPSCC in our study compared with studies conducted in Western countries with much higher prevalence of HPV-associated OPSCC. In future clinical trials that strictly address HPV status in HNSCC, HPV testing should be performed in all cases to precisely identify an association of HPV with OPSCC and non-OPSCC regardless of p16 expression, especially in areas with low prevalence of HPV infection.

## Supplementary Information

**Additional file 1: Supplement 1.** Real-time PCR typing of HPV with AmoyDx High-risk Human Papillomavirus (HPV) Detection Kit. One reaction with three fluorescents (A:FAM-other 17 HPVs high risk, B: Cy5-HPV 16/18 and C: HEX-Internal control) were mixed in each reaction tube for identification of the 19 possible HPV high risk types in a real-time PCR setting, as described in the Material and Methods section (1: positive control, 2: no template control, 3: positive other 17 HPVs high risk sample, 4: positive HPV type 16 or 18 sample and 5: internal control of sample). **Supplement 2.** Baseline characteristics of non-oropharyngeal HNSCC and p16 status.

## Data Availability

The datasets used and/or analysed during the current study available from the corresponding author on reasonable request.

## Abbreviations

AJCC: American Joint Committee on Cancer
CRT: Chemoradiotherapy
CI: Confidence intervals
DNA: Deoxyribonucleic acid
ECOG: Eastern Cooperative Oncology Group
FFPE: Formalin-fixed paraffin-embedded
HNSCC: Head and neck squamous cell carcinoma
HR: Hazard ratios
HPV: Human Papilloma virus
IHC: Immunohistochemistry
ISH: In situ hybridization
NR: Not reached
OPSCC: Oropharyngeal squamous cell carcinoma
OS: Overall survival
PCR: Polymerase chain reaction
RNA: Ribonucleic acid
