# Supplementary material for: Discrepancy in p16 expression in patients with HPV-associated head and neck squamous cell carcinoma in Thailand: clinical characteristics and survival outcomes
Source: BMC Cancer. 2021 May 6;21:504. doi: 10.1186/s12885-021-08213-9 (PMC8101232; doi:10.1186/s12885-021-08213-9)
Supplement: Supplementary file 1 — Additional file 1: Supplement 1. Real-time PCR typing of HPV with AmoyDx High-risk Human Papillomavirus (HPV) Detection Kit. One reaction with three fluorescents (A:FAM-other 17 HPVs high risk, B: Cy5-HPV 16/18 and C: HEX-Internal control) were mixed in each reaction tube for identification of the 19 possible HPV high risk types in a real-time PCR setting, as described in the Material and Methods section (1: positive control, 2: no template control, 3: positive other 17 HPVs high risk sample, 4: positive HPV type 16 or 18 sample and 5: internal control of sample). Supplement 2. Baseline characteristics of non-oropharyngeal HNSCC and p16 status. [file 12885_2021_8213_MOESM1_ESM.docx]

**Supplement 1**. Real-time PCR typing of HPV with AmoyDx High-risk Human Papillomavirus (HPV) Detection Kit. One reaction with three fluorescents (A:FAM-other 17 HPVs high risk, B: Cy5-HPV 16/18 and C: HEX-Internal control) were mixed in each reaction tube for identification of the 19 possible HPV high risk types in a real-time PCR setting, as described in the Material and Methods section (1: positive control, 2: no template control, 3: positive other 17 HPVs high risk sample, 4: positive HPV type 16 or 18 sample and 5: internal control of sample).

**Supplement 2.** Baseline characteristics of non-oropharyngeal HNSCC and p16 status

|  | **p16-Positive**  **n = 34 (6.5%)** | **p16-Negative**  **n = 484 (93.5%)** | **p-value** |
| --- | --- | --- | --- |
| Median Age (range)  Age ≥65 | 64.5 (38-81)  17 (50) | 62 (20-95)  205 (42) | 0.384 |
| ECOG  0-1  ≥2 | 31 (91)  3 (9) | 440 (90)  51 (10) | 1.000 |
| Sex  Male  Female | 24 (71)  10 (29) | 353 (72)  138 (28) | 0.87 |
| Smoking  Never  Ever  Mean pack-year (+/-SD)  Missing | 14 (42)  19 (58)  13 (14)  1 | 286 (62)  173 (38)  16 (19)  25 | 0.588  0.387 |
| Site of primary tumor  Oral cavity  Larynx  Hypopharynx  Paranasal sinus  Unknown primary | 12 (35)  13 (38)  5 (15)  4 (12)  0 | 245 (50)  131 (27)  89 (18)  23 (5)  3 (1) | 0.168 |
| Histology grade  Well-differentiation  Moderately-differentiation  Poorly-differentiation  Undifferentiation | 6 (21)  15 (54)  3 (11)  4 (14) | 169 (39)  184 (43)  26 (6)  52 (12) | 0.271 |
| Stage at Diagnosis (AJCC 7^th^)  I  II  III  IVa/b  IVc | 6 (18)  7 (21)  6 (18)  14 (41)  1 (3) | 81 (17)  44 (9)  79 (16)  260 (53)  25 (5) | 0.229 |
| T-stage  1-2  3-4  Tx | 18 (53)  16 (47)  0 | 187 (38)  298 (61)  6 (1) | 0.202 |
| N-Stage  0  1  2  3 | 19 (56)  6 (18)  8 (24)  1(3) | 231 (47)  82 (17)  146 (30)  31 (6) | 0.672 |
